# Individual Differences in Relational Learning and Analogical Reasoning: A Computational Model of Longitudinal Change

**DOI:** 10.3389/fpsyg.2018.01235

**Published:** 2018-07-24

**Authors:** Leonidas A. A. Doumas, Robert G. Morrison, Lindsey E. Richland

**Affiliations:** ^1^Department of Psychology, University of Edinburgh, Edinburgh, United Kingdom; ^2^Department of Psychology, Loyola University Chicago, Chicago, IL, United States; ^3^Department of Comparative Human Development, University of Chicago, Chicago, IL, United States

**Keywords:** analogical reasoning, relational knowledge, inhibitory control, development, computational modeling, cognitive control

## Abstract

Children’s cognitive control and knowledge at school entry predict growth rates in analogical reasoning skill over time; however, the mechanisms by which these factors interact and impact learning are unclear. We propose that inhibitory control (IC) is critical for developing both the relational representations necessary to reason and the ability to use these representations in complex problem solving. We evaluate this hypothesis using computational simulations in a model of analogical thinking, Discovery of Relations by Analogy/Learning and Inference with Schemas and Analogy (DORA/LISA; Doumas et al., 2008). Longitudinal data from children who solved geometric analogy problems repeatedly over 6 months show three distinct learning trajectories though all gained somewhat: analogical reasoners throughout, non-analogical reasoners throughout, and transitional – those who start non-analogical and grew to be analogical. Varying the base level of top-down lateral inhibition in DORA affected the model’s ability to learn relational representations, which, in conjunction with inhibition levels used in LISA during reasoning, simulated accuracy rates and error types seen in the three different learning trajectories. These simulations suggest that IC may not only impact reasoning ability but may also shape the ability to acquire relational knowledge given reasoning opportunities.

## Introduction

Analogical reasoning, the process of representing information as systems of relationships and mapping between these representations, is ubiquitous in learning and discovery throughout the lifespan, and is part of what makes humans uniquely intelligent and adaptive (Gentner, 2003; Penn et al., 2008). Analogical reasoning may play a crucial role in childhood, serving as a cognitive-bootstrapping mechanism that enables children to make increasingly abstract inferences and generalizations (e.g., Gentner, 2003), and supporting learning across a wide range of educational domains (Richland and Simms, 2015). The mechanisms by which children’s analogical reasoning improve, however, are not well understood. In particular, little attention has been paid to the processes by which children develop the relational representations used for analogical reasoning.

Children’s cognitive-control resources have been implicated as one source of individual differences in relational representation and reasoning (see Morrison et al., 2004; Viskontas et al., 2004; Simms et al., 2018). Also described as executive function (EF) (Diamond, 2013), these resources refer to the ability to use selective attention to manipulate the contents of working memory, and are believed to include a variety of functions including inhibitory control (IC), updating, and shifting (Miyake et al., 2000; Banich, 2009). Cross-sectional studies have revealed that children who can solve analogies successfully make mistakes when the requirements for cognitive control are raised, either by increasing the requirements for controlling attention in the face of distraction, or increasing the complexity of the relations (Richland et al., 2006; Thibaut et al., 2010a,b). The difficulty of controlling attention to relations in the face of distraction has been identified across children from different cultural and linguistic backgrounds (Richland et al., 2010). Computational work simulating such cross-cultural data through a combination of knowledge and IC has provided support for the interpretation that knowledge is necessary but not sufficient for representing relations, and that these errors are due to low levels of resources for IC (Morrison et al., 2011).

However, a full theory of relational reasoning development must go beyond performance accuracy to provide a mechanism for developmental change over time. There is reason to believe that cognitive-control resources not only predict performance at a single time point (see Simms et al., 2018), but also may impact children’s growth in reasoning skill. An analysis of data from a large-scale longitudinal study found that children’s performance at school entry on an IC task (Children’s Stroop; Gerstadt et al., 1994), and an EF task (Tower of Hanoi) both predicted distinct variance in children’s analogical skill, and more interestingly, their growth in analogical skill from school entry to adolescence (Richland and Burchinal, 2013). This relationship held even when controlling for environmental factors (e.g., parental education, SES, gender), as well as short-term memory, sustained attention, knowledge measures, and analogy skill at third grade. This pattern of change suggests that early EF skills play an important role in shaping children’s trajectory of learning reasoning skills.

### Testing EF As a Mechanism Underpinning Relational Reasoning Growth

The current paper reports computational simulations that test a mechanism by which early IC resources could alter the trajectory by which children’s reasoning develops through the course of children’s reasoning opportunities. We simulated data from one of the few longitudinal studies on the development of analogical reasoning (Hosenfeld et al., 1997a,b). Our aim was to explore how relational knowledge and variations in children’s IC could predict children’s rate of reasoning development over a series of repeated opportunities to solve geometric analogies. We focus in particular on the interplay between the learning of relational representations and individual differences in IC.

#### Behavioral Data on Reasoning Change Over Time

**FIGURE 1:**
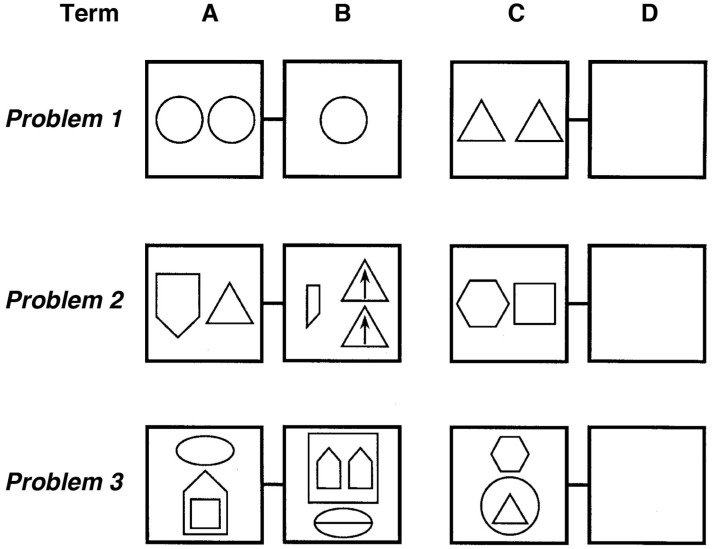
Hosenfeld et al. (1997a) developed a geometric analogy task with problems of varying complexity created using relations familiar to children (e.g., above/below, inside, halving or duplication, rotation). Examples of open-ended geometric analogy items of low (Problem 1), intermediate (Problem 2), and high level of difficulty (Problem 3). The D-term has to be filled in by the subjects (figure adapted from the Hosenfeld et al., 1997a).

In the original study (Hosenfeld et al., 1997b), 80 children aged 6–7 years, sampled randomly from the larger school sample available, solved 20 geometric analogy problems. Seventy-one of these children’s data were usable and were included in the final analyses. The geometric analogy problems tested children’s ability to identify and map five common relations between simple shapes including: adding an element, changing size, halving, doubling, and changing position repeatedly over eight testing sessions (**Figure 1**).

The period of testing ranged from 140 to 161 days, (mean 153, *SD* = 7.32) and the interval between the test sessions ranged from 13 to 35 days, with these being held constant across participant (the longest interval was between Sessions 2 and 3, when there was a school holiday). Participants were in regular school outside of this study, with no explicit training in relation to geometric analog ies. These were sessions in which participants solved problems and were given feedback, so in some ways these were both testing and training sessions.

The measure was originally designed by randomly combining six basic geometric shapes and five transformations in different ways to create 12,150 problems. The authors used the difficulty metric (Difficulty = 0.5 × Elements + 1 × Transformations) to select problems for a large norming project (Hosenfeld et al., 1997a). Twenty of these problems were then selected for use in the longitudinal study to represent a range of difficulty both with respect to the difficulty metric and actual child performance.

During testing, children solved A:B::C:D problems in which they had to infer the missing D term in order to construct a valid analogy. **Figure 1** provides three examples of these geometric analogy items in increasing difficulty, showing duplication (top line), halving/duplication and “inside” (middle line), and an above/below/inside set of transformations (bottom line).

On each testing occasion, the children were first given practice time. This included naming and drawing the basic geometric shapes that would be part of the relational problems. They were then told they would be solving puzzles and completed three practice analogies with the experimenter. The following instruction was provided: “These two boxes belong together (point to A and B), and those two boxes belong together (point to C and D). These two ones (A and B) belong together in the same way as those ones (C and D) do. Do you know what the solution is?” (Hosenfeld et al., 1997a, p. 375).

Twenty test items were then presented during each session, in which children were instructed to draw the missing piece for each problem and were provided with feedback following errors. The problems within each session varied in complexity based on changes in the number of relationships needed to characterize the A:B transition. The internal consistency was adequately high between items within each testing session, with alphas ranging from α = 0.87–0.91, using the standard that above a 0.7 is adequate. A Mokken scale analysis (Mokken, 1971) revealed monotone homogeneity and double monotonicity, allowing the authors to determine that the items and the subjects could reliably be ordered on a common dimension of difficulty. Thus, based on these difficulty ratings, parallel tests were constructed and validated to show comparable difficulty across the eight sessions.

Researchers recorded accuracy rates, time to solution, and types of errors made. These data were used to examine the trajectory of children’s analogical reasoning over the course of the study. Children’s performance was then fitted to parameter estimates of performance that reflected the proportion of analogical (versus non-analogical) responses as a function of test session. Modeling these parameters revealed three linear trends, the three learning profiles which will be simulated in this manuscript: (1) *Non-analogical reasoners*, who solved the majority of problems non-analogically throughout all sessions, (2) *Transitional reasoners*, who moved from solving problems largely non-analogically to solving problems largely analogically, and (3) *Analogical reasoners*, who solved the majority of problems analogically throughout the treatment. The reasoning accuracy results for the three groups of children over time are shown in **Figure 7**.

The data from Hosenfeld et al. (1997b) study are informative, and they raise a challenge of interpretation. One cannot fully explain these three trajectories by access to learning opportunities, since all children were exposed to the same number of training instances – though no data were provided in the original manuscript regarding whether children took advantage of training from all instances provided. Further, children’s initial skill-based starting point is not fully predictive either, since one group started low and ended high, and another group started low and ended low. Cognitive maturation of growth in EF capacity is a similarly unsatisfactory explanation. While some EF growth over the period of 6 months might be expected, there is no reason to expect three different yet systematic patterns of EF growth that would explain these three performance trajectories.

#### Current Simulation Study Aims

In the present study we use computational simulations of these data to argue that (1) differences in IC EF resources may explain initial differences in reasoning, but (2) they also help to explain differences between the three groups in their ability to learn relational representations necessary for reasoning over repeated learning opportunities. Thus, while all children received the same number of learning opportunities during the eight training sessions, the level of structure they identify in the problem inputs may increase or decrease their likelihood of successfully reasoning analogically with these representations over time. Furthermore, the rate at which they learn is constrained by their IC EF resources. The interaction of processing ability and learning representations produces a more complete picture of the development of analogical reasoning then either factor independently.

To assess this hypothesis, we examine learning patterns for a model with three levels of IC (high, medium, and low) and three levels of prior knowledge (after the first 100 learning trials, second and third 100 learning trials). Our aim is to best explain the three learning trajectories identified in the Hosenfeld et al. (1997b) data, and we find that an integration of IC and prior knowledge as described provide the best simulation.

### Computational Models of Analogical Reasoning

Computational models of analogical reasoning provide a unique window into the plausible cognitive underpinnings of relational reasoning, and here enable us to test correlations between the behavioral data and performance in a constrained system (see French, 2002). We use Discovery of Relations by Analogy (DORA; Doumas et al., 2008) as a model of how structured relational representations are learned from unstructured inputs, and Learning and Inference with Schemas and Analogy (LISA; Hummel and Holyoak, 1997, 2003) as a model of human relational reasoning, to simulate Hosenfeld et al. (1997b) results, and to explore the interactions between maturation-based inhibition levels and learning opportunity cycles.

Inhibition is critical for several aspects of LISA and DORA’s operation (see Knowlton et al., 2012). Reciprocal inhibition is fundamental to establishing the oscillations responsible for relational binding in LISA/DORA which occur via temporal synchrony or systematic asynchrony (see Hummel and Holyoak, 1997, 2003; Doumas et al., 2008). In fact, this property of inhibition is responsible for the models’ intrinsically limited working-memory capacities (see Hummel and Holyoak, 2003, Appendix A). However, inhibition is also important at another level of processing.

As discussed previously IC is critical during analogical reasoning to reduce interference from competing concepts sharing perceptual or semantic similarity with elements of the current information being considered in the analogy. Likewise other irrelevant relations present either in the source or a potential target may even interfere. Activation spreads between related concepts in the model with the most active units eventually entered working memory and thus being available for relational learning or reasoning. In order to keep focus on the critical relations under consideration in the source, the models postulate top-down lateral inhibition of propositions tagged as low in goal-relevance which helps prevent these propositions from entering the focus of attention in working memory.

Previously, we have successfully used changes in this top-down lateral inhibition in LISA’s working-memory system to explain cross-sectional variations in analogical reasoning. We simulated the developmental progression (from age 3 to 14 years) in children’s ability to handle increases in relational complexity and distraction from object similarity during analogical reasoning by varying IC (Morrison et al., 2011). In addition, we have modeled cross-cultural differences in analogy performance (Richland et al., 2010), considered to be the result of differences in relational knowledge accretion, via changes to the hand-coded representations used in LISA (Morrison et al., 2011).

In the current study we avoid hand coding of propositional structures. Instead, we use DORA to simulate children’s ability to learn spatial relations over time, allowing relational learning patterns to be part of the investigation. We then use those representations in LISA to simulate geometric analogy accuracy and types of errors. By doing so, we are able to model the trajectory of knowledge accretion as well as reasoning ability. Importantly, we manipulate top-down lateral inhibition (via changes to a parameter for this type of inhibition in both models) to simulate individual differences. We argue that IC is fundamental not only to the ability to reason relationally, but also to the ability to learn relations in the first place.

## Materials and Methods

### Overview of LISA/DORA Model

**FIGURE 2:**
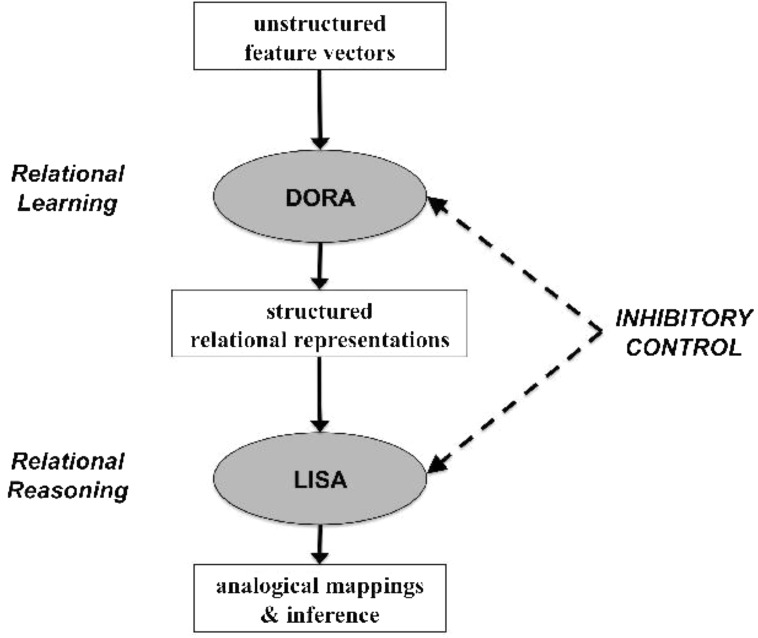
Schematic illustration of how DORA and LISA work together to enable relational learning and reasoning.

In this section we describe the LISA (Hummel and Holyoak, 1997, 2003) and DORA (Doumas et al., 2008) models in broad terms (see **Figure 2**). Our goal is to highlight the main processing features of the models and their core theoretical claims. Knowlton et al. (2012) provide another useful, brief description of the LISA architecture. The most complete descriptions of the models may be found in their original reports (Hummel and Holyoak, 1997, 2003; Doumas et al., 2008).

Both LISA and DORA (DORA is a direct descendent and generalization of LISA) are symbolic connectionist models. However, unlike traditional connectionist networks (e.g., McClelland, 2010), LISA and DORA solve the binding problem (the problem of reconciling which properties correspond to which object in a situation with two or more objects. For example, to represent that a red square is next to a blue circle, the system must be able to bind the square to the property of red and the circle to blue), and so can process structured (i.e., symbolic) representations (see Doumas and Hummel, 2005, 2012). LISA uses structured representations of relations (represented as predicates) and their arguments to make analogies, induce schemas, and perform relational generalization. While LISA assumes a vocabulary of representations provided by the modeler, DORA provides an account of how the structured predicate representations used by LISA can be learned from unstructured representations of objects in the first place (i.e., flat feature vector representations of objects without predicates; see below).

We begin by describing the representations that DORA starts with and those that it eventually learns. We then describe how DORA learns these knowledge structures from experience. Finally, we describe LISA’s mapping and generalization procedures. Both DORA’s learning and LISA’s mapping and generalization procedures play central roles in the simulations we report in this paper.

#### Knowledge Structures and Representational Form

**FIGURE 3:**
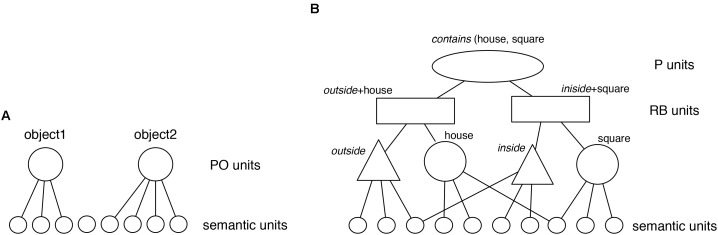
Representation of **(A)** predicates or objects in DORA and a **(B)** proposition in LISA.

Discovery of Relations by Analogy begins with objects represented as flat feature vectors (**Figure 3A**). That is, objects are represented as in conventional distributed connectionist systems as patterns of activation in a set of units. These initial representations are holistic and unstructured (see Doumas and Hummel, 2005). In the current simulations, these feature vectors are created by the modeler (as described in the “Simulations” section). However, in more recent work we have extended DORA to work from pixel images (e.g., Doumas et al., 2017a,b). Before describing how DORA learns structured predicate representations of object properties and relations from these initial representations; however, we will describe the end state of that learning. Specifically, we now describe knowledge representation in LISA (and by extension in DORA *after* it has learned).

Relational structures in the model are represented by a hierarchy of distributed and localist^1^ codes (**Figure 3B**), in a format defined as “LISAese” (see Hummel and Holyoak, 1997). At the bottom, “semantic” units represent the features of objects and roles in a distributed fashion. Semantic units don’t actually have any necessary meaning. They are simply properties of the perceptual stimulus that are detectable by the system (e.g., location on the *y*-axis, being cone shaped). As we discuss below, for the purposes of LISA and DORA, exactly what these semantic units code is not important. All that is necessary is that there are aspects of perceptual stimuli that are consistently detectable by the system (e.g., that when encoding two red objects, the same semantics – or set of semantics – responds to their hue). At the next level, these distributed representations are connected to localist units termed POs (for Predicate-Object) that represent individual predicates (or roles) and objects. One layer up, localist Role-Binding units (RBs; alternatively called “subpropositions”) link object and relational role units into specific role-filler pairs. At the top of the hierarchy, localist P (Proposition) units link RBs into whole relational propositions.

Considering the house object containing the *square* in Problem 3, Term A (**Figure 1**), the proposition *contains* (house, square) is represented by PO units (triangles and large circles in **Figure 3**) to represent the relational roles *outside* and *inside*, and the objects house and square. Each of these PO units is connected to semantic units coding their semantic features. RB units (rectangles) then conjunctively code the connection between roles and their fillers (one RB connects house to *outside*, and one connects square to *inside*). At the top of the hierarchy, P units (oval) link sets of RBs into whole relational propositions. A P unit conjunctively codes the connection between the RBs representing *outside* (house) and the RB representing *inside* (square), thus encoding the relational proposition *contains* (house, square).

Note that all of these units are simply connectionist nodes in a layered network. While we use different names for units at different layers, and use different shapes to specify different units in our figures, we do so only for the purposes of more efficient exposition. There is nothing inherently different about PO units or RB units other than they are in different layers of a neural network (much as different units might be in the input layer or a hidden layer of a feed-forward neural network). However, just as units in a hidden layer serve a different function in relation to a network’s behavior relative to units in the input layer, so units in the RB layer serve a different function than units in the semantic layer.

When a proposition enters working memory, role-filler bindings (i.e., a single role and it’s argument) must be represented dynamically on the units that maintain role-filler independence (i.e., POs and semantic units; see Hummel and Holyoak, 1997). In DORA (and its instantiation of LISA), roles are dynamically bound to their fillers by systematic asynchrony of firing. DORA’s working memory is composed of a driver [or the current focus of attention, akin to a “target” in analogical research (see Gentner, 1983)] and the recipient [akin to active memory per Cowan (2001) and akin to a “base” or “source” in analogical frameworks (see Gentner, 1983; Holyoak and Thagard, 1989)]. As a proposition in the driver becomes active, bound objects and roles fire in direct sequence. Binding information is carried in the proximity of firing (e.g., with roles firing directly before their fillers). Using the example in **Figure 3**, in order to bind *outside* to house and *inside* to square [and so represent *contains* (house, square)], the units corresponding to *outside* fire directly followed by the units corresponding to house, followed by the units for coding *inside* followed by the units for square.^2^

#### Learning Structured Representations in DORA

**FIGURE 4:**
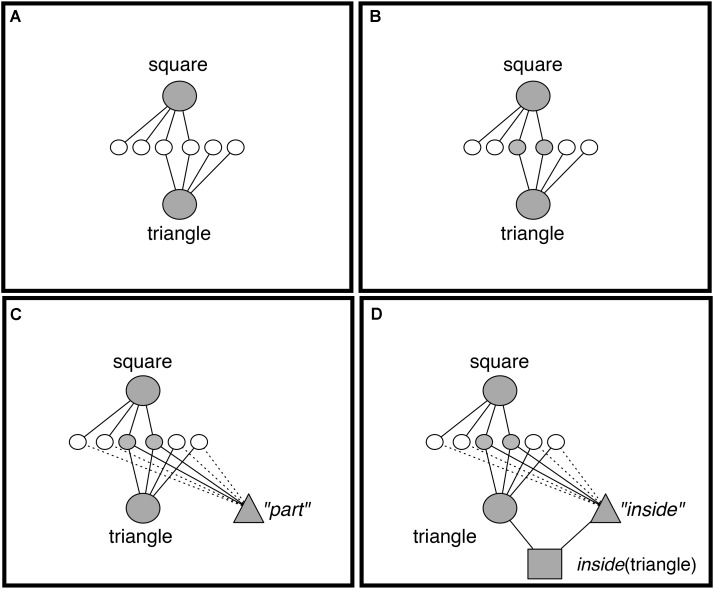
DORA learns a representation of *inside* by comparing a square that is inside some object to a triangle inside some object. **(A)** DORA compares square and triangle and units representing both become active. **(B)** Semantic units shared by the square and the triangle become more active than unshared semantics (darker gray). **(C)** A new unit learns connections to semantics in proportion to their activation (solid lines indicate stronger connection weights). **(D)** The new unit codes the featural overlap of the square and triangle (i.e., the role “*inside*”).

Discovery of Relations by Analogy is an account of how structured representations in the form used by LISA, LISAese representations, can be learned from unstructured examples. As noted above, DORA begins with representations of objects coded by simple flat feature vectors (**Figure 3A**). The 2d images in these analogy-training stimuli were coded as a set of semantics describing the perceptual characteristics of the geometric shapes (e.g., semantic units of a square), but we do not have a strong position on the level of semantic filtering that might impact such perceptual processes in everyday reasoning. We instantiate these representations as object token units attached to the semantic units of that object (**Figure 4A**). These initial representations are holistic and unstructured (in that an object’s semantics are active together as a mass; see e.g., Doumas and Hummel, 2005, 2012). DORA’s learning algorithm allows it to learn structured representations of specific subsets of an object’s semantics. Vitally, these representations function like predicates in that they are explicit and can take (i.e., be dynamically bound to) arguments.

Discovery of Relations by Analogy uses comparison to bootstrap its learning. When DORA compares two objects, then those objects become co-active (**Figure 4A**). As the compared objects pass activation to their semantic features, those properties shared by both objects receive twice as much input and become roughly twice as active as unshared semantic units (**Figure 4B**). DORA recruits a PO unit that learns connections to the active semantics via simple Hebbian learning. Accordingly, the new PO learns stronger connections to the more active (shared) semantics, and weaker connections to the less active (unshared) semantics (**Figure 4C**). DORA also recruits an RB unit at the layer above the POs, which learns connections to the active POs via Hebbian learning (**Figure 4D**).

The result of this learning algorithm is that DORA acquires explicit representations of the shared properties of compared objects. For example, when DORA compares two red things it will learn an explicit representation of the property *red*, and if DORA compares two objects that are containers, it will learn an explicit representation of the property *container*.^3^ Importantly, these new representations function like single-place predicates: they can be bound to arguments (via asynchronous binding; see above), they specify properties of the arguments to which they are bound (see Doumas et al., 2008; Doumas and Hummel, 2012), and they support symbolic operations such as structure mapping (see Hummel and Holyoak, 1997; Doumas et al., 2008) and relational generalization (Hummel and Holyoak, 2003).

**FIGURE 5:**
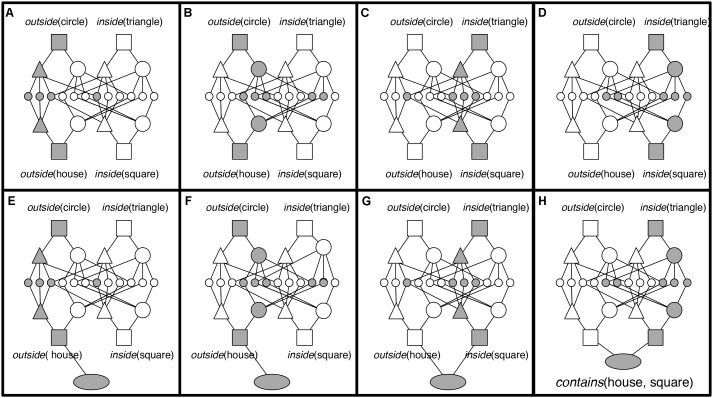
DORA learns a representation of the whole relation *contains* (house, square) by mapping *outside* (circle) to *outside* (house) and *inside* (triangle) to *inside* (square). **(A)** The units coding *outside* fire; **(B)** the units for circle and house fire; **(C)** the units for *inside* fire; **(D)** finally, the units for triangle and square fire. **(E–F)** DORA recruits a P unit that learns connections to the active RB unit [the RB coding for *outside* (house)] in the recipient. **(G, H)** The P unit learns connections to the active RB unit in the [the RB coding for *inside* (square)]. The result is a structure coding for *contains* (house, square).

Comparison underlies DORA’s ability to learn functional single-place predicate representations, and comparison also allows DORA to learn representations of whole relational structures (**Figure 5**). If multiple role-filler sets enter DORA’s WM together, the model can map each set onto the other. For example, if DORA compares the circle containing the triangle in **Figure 1** (Problem 3, Term C) to the house containing the square (Problem 3, Term A), it could map *outside* (circle) to *outside* (house) and *inside* (triangle) to *inside* (square). This process leads to a distinct pattern of firing over the units composing each set of propositions [i.e., the RB units of *outside* (circle) fire out of synchrony with those of *inside* (triangle) while the RB units of *outside* (house) fire out of synchrony with those of *inside* (square)]. This pattern of oscillating activation over sets of units (with co-occurring role-filler pairs firing in sequence) acts as a signal to DORA to recruit a P unit, which learns connections to active RBs via Hebbian learning. The result is that the new P unit links co-occurring role-filler sets, and results in a rudimentary representation of relations [here *contains* (object1, object2)]. Importantly, this kind of relational representation, in which a relation is composed of linked sets of its roles, is a full fledged multi-place relational structure capable of the same sorts of operations and inferences as traditional multi-place relations (e.g., predicate calculus; Doumas and Hummel, 2005, 2012; Doumas et al., 2008).

#### Mapping and Relational Generalization in LISA

In LISA/DORA, representations are divided into two mutually exclusive banks of units: a *driver* and one or more *recipients*.^4^ The driver is the current focus of attention (i.e., what LISA/DORA is thinking about at the present moment), and the recipient is analogous to active memory in Cowan (2001) terms (i.e., items primed from long-term storage, which can be potentially compared to items in the driver). The driver and recipient communicate via the semantic feature units, which are shared by both sets. Specifically, items in the driver become active and pass activation to the semantic feature units, which activate units in the recipient. Units in the recipient then compete via lateral inhibition to respond to the pattern of firing imposed on semantic units by units in the driver. Lateral inhibition is used here to refer to the type of inhibition which DORA/LISA uses within relational structures to reinforce those structures. This type of lateral inhibition has also been referred to as driver/recipient inhibition (Morrison et al., 2011) and also as “top-down inhibition” (Knowlton et al., 2012). Manipulation of the parameter controlling this type of inhibition was previously used to simulate developmental effects in analogical reasoning (Morrison et al., 2011) and analogy performance in patients with damage to prefrontal cortex (Morrison et al., 2004). It is assumed that this type of inhibition is a particularly important function of the prefrontal cortex [see Knowlton et al. (2012) for a detailed discussion of the role of inhibition in relational reasoning and how DORA/LISA implements this]. This lateral inhibition is not to be mistaken for a more general type of inhibition found throughout the nervous system and essential for implementing oscillatory dynamics (see Hummel and Holyoak, 2003, Appendix A).

Structured representations created during relational learning in DORA can be mapped using LISA’s mapping algorithm (Hummel and Holyoak, 1997) with minor modifications described in Doumas et al. (2008). LISA/DORA learns which elements in the driver and recipient correspond by building mapping connections (via Hebbian learning) that keep track of when these elements are active simultaneously. Importantly, LISA/DORA can make map role-filler pairs even when the fillers have absolutely nothing in common. For example, if LISA/DORA encounters two objects that are completely featurally independent, but are both bound to the *higher* role (i.e., both objects are higher than some other thing), it can map them. This capacity allows LISA/DORA to make mappings based solely on the relational properties of objects (see e.g., Hummel and Holyoak, 2003; Doumas et al., 2008). At the same time, LISA/DORA can make incorrect analogies if there is no relational correspondence.

When augmented with the capacity for self-supervised learning (Hummel and Holyoak, 2003; Doumas et al., 2008; described below), LISA’s mapping algorithm allows for analogical inference. To illustrate, consider how LISA/DORA solves an inference problem such as the third problem in **Figure 1**^5^. The A and B terms are in the driver and the C term is in the recipient. As the proposition coding for A term, *contains* (house, square), becomes active in the driver, it activates and consequently maps to the units coding for *contains* (circle, triangle) in the recipient. Specifically, the units coding for *outside* (house) in the driver activate and map to the units coding for *outside* (circle) in the recipient, and the units coding for *inside* (square) in the driver activate and map to the units coding for *inside* (triangle) in the recipient.

Then when the B term, *contains* (square, shield) becomes active in the driver, there are no corresponding units for it to map to in the recipient. As the representation of the C term in the recipient is already mapped to the representations of the A term in the driver (and the C term is the only item in the recipient), the representation of the B term is left with nothing to which it corresponds. This situation, in which items in the driver have no elements in the recipient that they can activate (because all recipient elements are already mapped to other driver elements), triggers the self-supervised learning algorithm in LISA/DORA. During self-supervised learning, active units in the driver prompt LISA/DORA to recruit matching units in the recipient (i.e., an active RB unit in the driver prompts recruitment of an RB unit in the recipient). Continuing the example, as units coding for *outside* (square) in the B term become active in the driver, LISA/DORA recruits RB and P units in the recipient to match the active RB and P units in the driver. The new recruited P unit in the recipient learns connections to active recipient RB units, and newly recruited RB units learn connections to active PO units via Hebbian learning. This is a strictly layered model. The functional result of this unit-based recruitment and Hebbian learning is that LISA/DORA infers a representation of *outside* (triangle) in the recipient, which corresponds to the representation of *outside* (square) in the driver. An analogous sequence occurs when *inside* (house) fires in the driver and LISA/DORA infers *inside* (circle) in the recipient. Thus, LISA/DORA completes the D term in a problem via analogical inference, inferring a representation of *contains* (triangle, circle) in the recipient.

##### The role of inhibition in DORA/LISA

Of particular importance to the present simulations, inhibition plays a role in the selection of items to enter working memory because selection is a competitive process. As noted above, inhibition is conceptualized here not at the low level of neuronal firing, nor operationalized at the high level of overall brain activity, but rather as part of the attentional control aspects of the working memory system that would control what representational information enters active working memory. More specifically, propositions in the driver compete to enter into working memory on the basis of several factors, including their pragmatic centrality or importance, support from other propositions that have recently fired, and the recency with which they themselves have fired. Reduced driver inhibition results in reduced competition and more random selection of RBs to fire. The selection of which RBs are chosen to fire, and in what order, can have substantial effects on DORA/LISA’s ability to find a structurally consistent mapping between analogs. It follows that reduced driver inhibition, resulting in more random selection of propositions into working memory, can affect DORA/LISA’s ability to discover a structurally consistent mapping.

The role of inhibition in the activity of a recipient analog is directly analogous to its role in the activity in the driver. Recipient inhibition causes units in the recipient to compete to respond to the semantic patterns generated by activity in the driver. If DORA/LISA’s capacity to inhibit units in the recipient is compromised, then the result is a loss of competition, with many units in the recipient responding to any given pattern generated by the driver. The resulting chaos hampers (in the limit, completely destroys) DORA/LISA’s ability to discover which units in the recipient map to which in the driver. In short, inhibition determines DORA/LISA’s working memory capacity (see Hummel and Holyoak, 2003, Appendix A; Hummel and Holyoak, 2005), controls the model’s ability to select items for placement into working memory, and also regulates its ability to control the spreading of activation in the various recipient analogs (see Knowlton et al., 2012). As such, inhibition is critical for the model’s ability to favor relational similarity over featural similarity. Relevant representations were sampled randomly from LTM. Relevance was defined as being strongly connected (weight above 0.9) to semantics defining a specific relation.

This conception is highly complementary to behavioral models suggesting IC in EF contributes to reasoning performance by enabling reasoners to inhibit rules used previously in favor of current goal requirements (e.g., Zelazo and Frye, 1998; Zelazo et al., 2003). Thus, we hypothesized that differences between the three groups of children in Hosenfeld et al. (1997b) study were at least partially a product of differences in IC. We simulated these differences in DORA/LISA by varying levels of lateral inhibition. In DORA/LISA, inhibition is critical to the selection of information for processing in working memory. Specifically, inhibition determines the intrinsic limit on DORA/LISA’s working-memory capacity (see Hummel and Holyoak, 2003, Appendix A), controls its ability to select items for placement into working memory, and also regulates its ability to control the spreading of activation in the recipient [see Knowlton et al. (2012) and Morrison et al. (2004, 2011) for discussions of these latter roles]. We have previously used this approach in LISA to simulate patterns of analogy performance in a variety of populations with lesser working-memory capacity including older adults (Viskontas et al., 2004), patients with damage to prefrontal cortex (Morrison et al., 2004), and young children (Morrison et al., 2011).

### Simulations

We simulated Hosenfeld et al. (1997b) results in two steps (**Figure 2**). In the first step we used DORA’s relation-learning algorithm to learn representations of the transformations used in the geometric analogy problems. In our simulations, DORA began with representations of 100 objects attached to random sets of semantic units (chosen from a pool of 1000). We then defined five transformations [the same as those used by Hosenfeld et al. (1997b): adding an element, changing size, halving, doubling, and changing containment]. Each single-place predicate transformation (adding an element, changing size, halving, doubling) consisted of two semantic units, and the relational transformation (changing containment) consisted of two roles each with two semantic units (i.e., for the *contains* relation, both the roles inside and outside were each defined by two specific semantic units). Again, as noted above, these semantic units had no actual content. Rather, they represented our assumption that there are invariant properties of objects and transformations that are detectable by the perceptual system. Our goal in this first simulation was simply to demonstrate that DORA could isolate and learn explicit representations of invariant properties during completely unstructured training.

Each of the 100 objects was attached to the semantics of between two and four transformations chosen at random. If an object was part of a relational transformation, it was attached to the semantics of one of the roles, chosen at random. For example, object1 might be attached to the semantics for *doubled* (a single-place transformation) and *inside* (one role of the relational transformation, *contains*).

We presented DORA with sets of objects selected at random, and allowed it to compare the objects and learn from the results (applying DORA’s relation-learning algorithm). As DORA learned new representations it would use these representations to make subsequent comparisons. For example, if DORA learned an explicit representation of the property *double* by comparing two objects both attached to the semantics of *double*, it could use this new representation for future comparisons. On each trial we selected between two and six representations and let DORA compare them and learn from the results (i.e., perform predication and relation-learning routines). We assume that this act of inspection and comparison is similar to what happens when children encounter the geometric analogy problems and have to consider how the various elements are related (Gentner and Smith, 2013). Importantly, this training was completely unstructured and undirected (i.e., DORA randomly selected items from memory to reason about). We have demonstrated in previous work that DORA can learn under these unstructured conditions, and that learning improves markedly with more directed or more structured training (see Doumas et al., 2008). We have no doubt that the children in Hosenfeld et al. (1997b) study learned from their experience with the various versions of the geometric analogy task, and that taking the test over successive sessions served to structure their training somewhat. For the current simulations, however, we wanted to make as few assumptions about the learning environments of the children in the study as possible (given this information is, very understandably, absent from Hosenfeld et al., 1997b). As such, we chose to handicap ourselves and avoid making additional assumptions that would improve our overall ability to fit the data.

Moreover, we defined three groups for the purposes of the simulation as determined by a range of lateral inhibition values. We ran 100 simulations for each group. During each simulation we chose an inhibition level from a normal distribution, with a mean of 0.4 for the low inhibition group, 0.6 for the medium inhibition group, and 0.8 for the high inhibition group (each distribution had a *SD* = 0.1). These were selected to evaluate the hypothesis that these three groups would show the same pattern of analogy performance trajectories as in the behavioral data. This would mean that the high inhibition group would align with the Analogical group, beginning and continuing to generate analogical solutions. The middle inhibition group would simulate the Transition trajectory, beginning non-relational and ending analogical, and the low inhibition group would simulate the Non-Analogical group, who begin and end non-analogical. We chose to simulate groups using a distribution of inhibition scores in order to match our assumption that the learning groups from Hosenfeld et al. (1997b) study were not completely homogeneous in their inhibitory abilities. Our decision once again served to handicap the precision of our simulations by adding some noise, but there was almost certainly some natural variability in the inhibitory abilities of the children in the initial study, and we wanted our simulations to reflect this variability.

For the low-knowledge condition, simulations were run with 800 learning trials, and we checked the quality of the representations DORA had learned after each 100 learning trials. Quality was calculated as the mean of connection weights to relevant semantics (i.e., those defining a specific transformation or role of a transformation) divided by the mean of all other connection weights +1 (1 was added in the denominator to keep the quality metric bound between 0 and 1). For the high-inhibition, high-knowledge condition we extended the simulations to 1000 learning trials and sampled the representations after 300–1000 trials. The reason for the different knowledge conditions was to test our hypothesis that children in the group that started high and stayed analogical not only had higher inhibitory resources, but also came into the study with a higher quality of relational representations. In brief, our goal was to test whether starting at a higher knowledge state in tandem with increased inhibitory resources would provide a closer fit to the analogical throughout group’s data than increased inhibitory resources in isolation.

**FIGURE 6:**
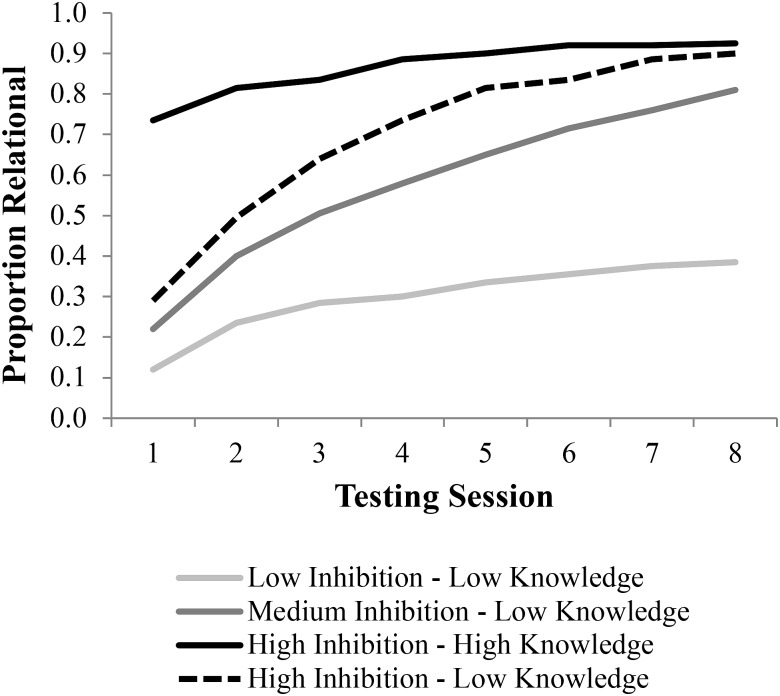
Simulation of relational learning in DORA. DORA’s relational learning algorithm was run at either low (0.4), medium (0.6), or high (0.8) lateral inhibition levels for 100–800 iterations to generate representations used in LISA for the low-knowledge condition. For the high-knowledge version a high (0.8) lateral inhibition level was used for 300–1100 iterations.

**FIGURE 7:**
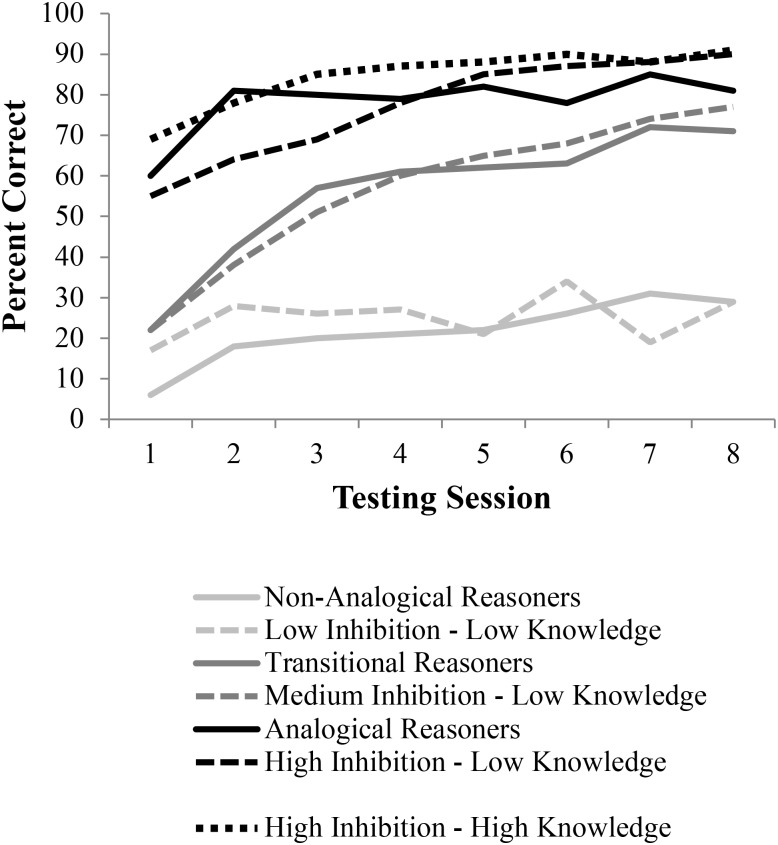
Results from children (Hosenfeld et al., 1997b) and LISA simulations. Simulation results were obtained by allowing LISA to make analogical inferences using the representations generated in DORA (**Figure 6**). The three performance groups of children were simulated by using three different levels of lateral inhibition in both DORA and LISA. Solid lines depict the data from “Hosenfeld et al. (1997b)” while the dashed lines are the simulated data.

**Figure 6** provides a summary of results from Part 1 of the simulation. While all groups did learn, learning was obviously improved with higher levels of inhibition. In addition, learning was much faster for the higher inhibition group. The simulation data are presented using the eight testing trials in the behavioral data to frame intervals and to allow for comparing the simulations to the empirical data, which will be added in **Figure 7**.

In the second part of the simulation we passed the representations DORA learned during the first part of the simulation to LISA, which then simulated solving the geometric analogy problems. Thus, unlike LISA simulations we have performed previously to account for developmental changes (e.g., Morrison et al., 2011), relational knowledge representations were not hand-coded, but rather were generated automatically by DORA. We created problems of varying difficulty to capture the range of difficulty used in the Hosenfeld et al. (1997b). Thirty percent of problems were hard problems consisting of three transformations (one-third had two binary transformations and one unary transformation; one-third had one binary transformation and two unary transformations; and one-third had three unary transformations). Thirty-five percent were medium difficulty problems consisting of two transformations (one-third had two binary transformations; one-third had two unary transformations; and one-third had one binary and one unary transformation). Lastly, 35% were easier problems with only one transformation per problem (half had one binary transformation while the other half had one unary transformation).

We simulated all eight of the testing phases in the Hosenfeld et al. (1997b) study. Each testing phase consisted of 20 trials. On each trial we presented LISA/DORA with the A and B terms in the driver and the C term in the recipient. The A, B, and C terms were represented as object POs each attached to four random semantics, and bound to PO predicate units identifying the transformations in which they were involved. Importantly, the PO units identifying the transformations (as well as the RB units linking predicate and object POs, and P units linking RBs) were representations that DORA had learned during the first part of the simulation. For example, if the A term was a shield inside a square, we represented that with the LISEese proposition *contains* (square, shield), with a PO representing *square* bound to a PO representing *outside* (where *outside* was a PO that DORA had learned during the first part of the simulation), and a PO representing *shield* bound to a PO representing *inside* (where inside was a PO that DORA had learned during the first part of the simulation). For the first testing phase for the low-knowledge groups we used the representations DORA had learned after the first 100 learning trials as described above, for the second testing phase we used the representations DORA had learned after the first 200 learning trials, and so on. For the high-knowledge groups, we used the representations DORA had learned after the first 300 learning trials for the first testing phase, the representations learned after the first 400 learning trials for the second testing phase, and so forth. In each knowledge condition we treated the level of lateral inhibition as maturational, and thus used the same levels as used for the learning phase for each group [0.4 low, 0.6 medium (or transitional), 0.80 high; each with ± 0.1 SD distribution]. We used the same level because the interval from the beginning and end of the training study was a short period of time in the developmental trajectory of children’s EFs, so we would expect EFs not to change within an individual in that period of time.

As noted directly above, using slightly more advanced representations (the high-knowledge group) reflects the assumption that children with higher maturational IC are likely to have learned more about relations prior to beginning the study compared to children with lower maturational IC. Note that by starting testing with representations at 100 we assume that all children have some capacity for representing relations. This assumption is reflected in Hosenfeld et al. (1997b) data, in that low- and transitional-analogy group children started with similarly low scores in the first testing phase, whereas children in the high-analogy group started with much higher performance on the first testing phase. Thus, we examine whether relational knowledge and IC contributed differently to the behavioral data, which they do seem to do, though the combination of knowledge and IC best explains the growth trajectories. This supports the claim that these are likely to co-vary but they each provide additional contributions.

During test trials, LISA attempted to map driver and recipient propositions and make inferences about the missing D term. For example, if LISA mapped the A term in the driver to the C term, then when the B term fired LISA inferred the D term in the recipient. We took the inferred proposition in the recipient to be LISA’s answer on that trial.

As is apparent from the learning trajectories plotted in **Figure 7**, DORA/LISA’s performance on the testing trials closely followed those of the children in Hosenfeld et al. (1997b) study. Just like the non-analogical children, DORA/LISA with a low lateral inhibition level performed poorly throughout. Like the transitional children, DORA/LISA with a medium lateral inhibition level started slow, but slowly improved. Finally, like the analogical children DORA/LISA with high lateral inhibition levels performed well virtually from the start and maintained good performance; however, additional relational knowledge coupled with high lateral inhibition levels appears to best fit the analogical performance group.

Importantly, the types of errors that DORA/LISA makes closely follow the types of errors made by each of the performance groups (**Table 1**). Specifically, like the non-analogical children, low-inhibition DORA tended to make errors based on featural association errors (e.g., objects in A, B, and C copied). Like transitional children, with medium inhibition, DORA tended to make featural/associative errors at the beginning, but these largely disappeared by the final session. Finally, like the analogical children, with high inhibition, DORA tended to make fewer errors overall, which further decreased over time, but these errors that did happen were a mix of associative and incomplete solutions.

**Table 1:** Solution Patterns in Children’s and DORA/LISA Simulations for the Three Learning Trajectories

	Non-analogical		Transitional		Analogical		
	Children	DORA/LISA (low knowledge)	Children	DORA/LISA (low knowledge)	Children	DORA/LISA (low knowledge)	DORA/LISA (high knowledge)
Analogical solution	21	25	56	57	78	77	85
Incomplete solution	21	17	28	26	18	17	13
Associative solution	58	58	16	17	3	6	2

*Children’s results based on data presented in **Table 5** (p. 384) of Hosenfeld et al. (1997b).*

Moreover, the kinds of problems that DORA “got wrong” at various inhibition levels seem highly in line with the kinds of problems that children seem to make errors on as they develop. While Hosenfeld et al. (1997b) do not give specific data on which problems the children tended to get wrong, a good deal of previous research has been done on children’s analogical development using cross sectional designs (see above; e.g., Richland et al., 2006). Generally, children tend to develop a capacity for solving simpler analogy problems first, and solve such problems consistently before they develop the capacity for solving harder analogy problems. For example, Richland et al. (2006) found that young children around the age of 3 years perform consistently above chance on simple analogy problems that require aligning pairs of elements across two pictures (e.g., a task that requires matching the cat in a picture of a dog chasing a cat, to the boy, in a picture of a mother chasing a boy). However, these same children perform very poorly when the task is made harder, either by adding distractor elements to one or both of the pictures, or by requiring integration across multiple relations (see above). Around age 7 years, children consistently solve problems either requiring relational integration or involving distractors, but perform less well on problems involving a distractor and requiring relational integration. Finally, by age 14 years, participants could consistently solve all the types of analogy problems tested. Similarly, DORA, across all inhibition levels, performed well on some classes of problems and less well on other classes. Specifically, low-inhibition DORA did consistently quite well on the easiest problem types, but quite poorly on the medium and hard problems. Medium inhibition DORA performed well on easy problems, made some headway on the medium problems, and did very poorly on the hard problems. High inhibition DORA was competent across all problem difficulties, but failed most consistently on the hard problems.

## Discussion

These simulations provide a mechanism by which resources for IC can account for children’s analogical reasoning development (Richland and Burchinal, 2013). We have previously argued that IC is an essential factor in understanding the development of analogical reasoning in children because changes in IC can explain both featural distraction and relational complexity effects during childhood (Morrison et al., 2011). However, a complete understanding of the development of analogy must also include the role of *relation learning* and the factors that impact the *growth* of relational knowledge over time. With these simulations we demonstrate that with learning opportunities the model, like children, moved from preferentially attending to featural information, to reasoning with relational representations. These simulations suggest that a child’s level of IC in working memory may play an essential role in determining their learning trajectory by modulating the noise through which children identify and train their relational representations. Thus, changes in IC during learning and reasoning help to explain the relational shift (Gentner and Rattermann, 1991) observed in young children’s relational reasoning.

Why does such a simple change in a single parameter have such a complex effect and thus explain so much? We theorize that this results from IC in working memory being important not only for relational reasoning but also for the process of learning relational representations, which occurs as children attempt to use relational reasoning. In the past, research in this area has focused more on the roles of IC during reasoning, and pre-existing knowledge. We have previously shown that greater IC in an individual can help a child avoid distraction from irrelevant information within relational representations (see Morrison et al., 2011). And, while it is frequently argued that analogical reasoning ability is tied to pre-existing knowledge of a domain and thus better structured representations (e.g., Gentner and Rattermann, 1991), these simulations show that regardless of the quality of prior knowledge representations, the ability to inhibit mappings due to non-relationally central aspects of representations may also rely on adequate IC. This means that an adult can form a valid analogy between two domains about which they know relatively little, allowing them to presumably improve their knowledge representations, whereas a 3-year-old child might know quite a bit about two domains yet still fail to inhibit a featural distractor when attempting to make an analogical mapping.

The second way that the IC parameter impacts performance is specifically tied to relational learning. In our model, IC is necessary for not only relational reasoning but also relational learning, an assumption supported by longitudinal studies showing that early EFs can predict children’s growth rates in analogy performance (Richland and Burchinal, 2013). Thus, the model posits that children with lower IC will learn relations less efficiently, satisficing based on featural similarities rather than noticing relational commonalities across representations, and thereby moving toward reasoning analogically. This means they may take more time to identify and abstract common relations across representations, and sometimes (as in the specific case of the non-analogical learners here), may not learn key relations despite multiple opportunities to abstract them from dirty representations.

The combination of these two factors results in our complex pattern of simulations. The simulations suggest that the children low in IC had difficulty building relationally precise representations, and also were less able to reason with these “dirty,” incomplete representations during reasoning, likely leading to more distraction. In contrast, children with high IC built relationally precise representations quickly and were also more tolerant of “dirty” representations, reasoning based on the relevant relational correspondences and minimizing errors based on irrelevant distractors. Our middle IC group operates at the perfect “teachable moment,” something akin to Vygotsky’s zone of proximal development (Vygotsky, 1978) – they possess just the right amount of IC to efficiently build relational representations which become sufficient during the training sessions to yield successful analogical reasoning.

One very important limitation of our current simulations stems from the kinds of problems used in the original Hosenfeld et al. (1997b) study, and in our simulations. Specifically, as has been argued previously (e.g., Thibaut and French, 2016), in the A:B::C:D tasks, the subject’s goal is to find the item (D) that matches (B) in the same way that (C) matches (A). The fact that (C) corresponds to (A) is given by the structure of the problem. In many tasks people draw analogies between situations without knowing these sorts of correspondences beforehand (e.g., Markman and Gentner, 1993; Richland et al., 2006). However, as argued above, we believe the longitudinal nature of the data collected by Hosenfeld and colleagues has a number of merits and that any full account of the development of relational reasoning needs to account for the findings that this study reveals. Particularly, (a) Hosenfeld et al. (1997a,b) is, to our knowledge, the only longitudinal study of analogical development with multiple repeated data collection points; (b) the difficulty of many of the problems used in the study makes solving them difficult even for adults; and (c) the (D) term was not given to the children to select (as is often the case with A:B::C:D analogy problems), but rather had to be generated in full. Finally, it is important to note that in our simulations we did not give DORA the (A)–(C) correspondence *a priori.* DORA had to discover the (A)–(C) correspondence via it’s mapping algorithm, and failure to do so made the generation of the (D) term all but impossible. As such, while our simulations are limited by the exclusive use of A:B::C:D type analogy problems in the original study, we do find the original study a very important piece in our current understanding of analogical development, and, therefore, hold that simulating the study is an important milestone for any account of the development of analogical thinking.

To conclude, while considerable effort has been directed at understanding how IC supports analogical reasoning, less attention has been given to the role of IC in its essential antecedent – relational learning. Appreciating the importance of IC during both relational learning and reasoning constitutes an important step toward understanding how relational learning develops and how it can contribute to successful analogical reasoning in children.

## Author’s Note

A preliminary report of these simulations was presented at the 31st Annual Conference of the Cognitive Science in Amsterdam, Netherlands.

## Author Contributions

The authors contributed equally to the conceptual development of the article. Dr. LD programmed the computer models and collected the simulation data. All authors each wrote various sections of the manuscript, and each edited the entire manuscript.

## Conflict of Interest Statement

The authors declare that the research was conducted in the absence of any commercial or financial relationships that could be construed as a potential conflict of interest.

## Footnotes

1 A localist representation uses a single node to represent a single concept. By contrast, a distributed representation uses a collection of nodes to represent a concept.

2 Asynchrony-based binding allows role and filler to be coded by the same pool of semantic units, which allows DORA to learn representations of relations from representations of objects (Doumas et al., 2008).

3 As noted above, the specific content of the units coding for a property are unimportant to DORA. So long as there is something common across the units representing a set of objects, DORA can learn an explicit representation of this commonality. For the purposes of DORA’s learning algorithm, all that matters is there is something invariant across instances of a *container* (which there must be for us to learn the concept), and that the perceptual system is capable of responding to this invariance (which, again, there must be for us to respond similarly across instances of containment in the world).

4 Mutually exclusive sets are necessary in order to perform comparison (see e.g., Falkenhainer et al., 1989; Holyoak and Thagard, 1989). Knowlton et al. (2012) describe how such sets can be instantiated in prefrontal cortex in a neutrally plausible fashion.

5 The problem is more relationally complex than the simple version we describe here; however, the same principles apply to the way LISA can solve the entire problem, including all of the nested relations.
